# Draft Genome Sequence of *Mycobacterium mucogenicum* Strain CSUR P2099

**DOI:** 10.1128/genomeA.01369-15

**Published:** 2015-11-19

**Authors:** Shady Asmar, Nicolás Rascovan, Catherine Robert, Michel Drancourt

**Affiliations:** Aix Marseille Université, URMITE, UMR 63, CNRS 7278, IRD 198, Inserm 1095, Faculté de Médecine, Marseille, France

## Abstract

*Mycobacterium mucogenicum* is a rapid-growing, nontuberculosis *Mycobacterium* species. The draft genome of *M. mucogenicum* CSUR P2099 comprises 6,210,127 bp exhibiting a 67.2% G+C content, 6,003 protein-coding genes, and 91 predicted RNA genes.

## GENOME ANNOUNCEMENT

*Mycobacterium mucogenicum* is a rapid-growing mycobacterium formerly known as a *Mycobacterium chelonae*–like organism (1, 2). *M. mucogenicum* was commonly implicated in outbreaks of opportunistic infections traced to contaminated water systems (3, 4) and contaminated hospital equipment (5–7). Catheter-related infections are the major form of *M. mucogenicum* opportunistic infection (8, 9), and *M. mucogenicum* is the leading mycobacterium to cause catheter-related bloodstream infections (9). However, respiratory tract, skin, soft tissue, and central nervous system infections due to *M. mucogenicum* have also been reported (10). We performed the whole-genome sequencing of *M. mucogenicum* CSUR P2099 in order to promote the development of advanced molecular tools for the detection and identification of this species.

*M. mucogenicum* CSUR P2099 was cultured in MGIT Middlebrook liquid culture (Becton, Dickinson, Le Pont-de-Claix, France) at 37°C in a 5% CO_2_ atmosphere. Genomic DNA was then sequenced by Illumina MiSeq runs (Illumina, San Diego, CA, USA) using a 5-kb mate-paired library. Reads were trimmed using Trimmomatic (11) and assembled using Spades version 3.5 (12, 13). Contigs were combined together by SSPACE version 2 (14) and Opera version 2 (15) and helped by GapFiller version 1.10 (16). This resulted in a draft genome consisting of seven scaffolds and seven contigs for a total of 6,210,127 bp and a G+C content of 67.2%. Noncoding genes and miscellaneous features were predicted using RNAmmer (17), ARAGORN (18), Rfam (19), PFAM (20), and Infernal (21). Coding DNA sequences were predicted using Prodigal (22), and functional annotation was achieved using BLASTp against the GenBank database (23) and the Clusters of Orthologous Groups (COGs) database (24, 25). The genome was shown to encode at least 91 predicted RNAs, including 6 rRNAs, 57 tRNAs, 1 tmRNA, and 27 miscellaneous RNAs. A total of 6,003 identified genes, including 3,678 (61.27%) encoding putative proteins and 1,884 (31.38%) assigned as hypothetical proteins, yielded a coding capacity of 6,145,630 bp (coding percentage, 98.96%). A total of 4,199 (69.9%) genes matched at least one sequence in the COGs database with BLASTp default parameters.

### Nucleotide sequence accession numbers.

The *M. mucogenicum* genome sequence has been deposited in EMBL under the accession numbers CYSI01000001 to CYSI01000007.
